# Supplementary material for: Onset, timing, and exposure therapy of stress disorders: mechanistic insight from a mathematical model of oscillating neuroendocrine dynamics
Source: Biol Direct. 2016 Mar 25;11:13. doi: 10.1186/s13062-016-0117-6 (PMC4807591; doi:10.1186/s13062-016-0117-6)
Supplement: Additional file 1 — The file provides appendices describing the mathematical details of our analysis. It includes discussions on nondimensionalization and parameter estimations used in our model. Analyses of the nullcline structure and the parameter space are also provided and the effect of stress onset timing on initial stress response is described in detail. Finally, a possible form of cortisol dependent I ext is illustrated with its implications on model behavior. (PDF 2099 kb) [file 13062_2016_117_MOESM1_ESM.pdf]

Additional File 1

Below, we provide appendices describing the mathematical details of our analysis.

Nondimensionalization

Our equations are nondimensionalized in a manner similar to that used by Walker *et al.*:

$$\begin{aligned} t &= d_O T, & c_s &= C_s/\bar{C}_s, & c &= \mu_R p_C d_O C, \\ a &= \mu_R p_C d_O^2 A, & r &= \mu_R p_C d_O R, & o &= \mu_R p_C p_A p_O d_O^3 O, \end{aligned} \quad \text{(A1)}$$

Here, c_s, c, a, r, o are the dimensionless versions of the original concentrations C_s, C, A, R, O, respectively. C_s is normalized by $\bar{C}_s$, which denotes the typical maximum amount of releasable CRH in the physiological range. Upon using these variables, the dimensionless forms of Eqs. 9-13 are expressed in Eqs. 14-18. The parameters q_i, p_i are dimensionless combinations conveniently defined to be analogous to those used by Walker *et al.*:

$$\begin{aligned} t_c &= d_O T_C, & t_d &= d_O T_d, & q_0 &= p_C/(\mu_R p_R), \\ q_2 &= d_C/d_O, & p_2 &= \mu_R^2 p_R^2 p_A p_O/(d_O^4 K_A), & p_3 &= d_A/d_O, \\ p_4 &= p_C^4 p_A p_O d_O^8 K_R^2/\mu_R, & p_5 &= 1/\mu_R, & p_6 &= d_R/d_O. \end{aligned} \quad \text{(A2)}$$

Using these scalings, we arrive at the dimensionless Eqs. 14-19.

Parameter estimates

Many of the numerous physiological parameters in our model can be estimated or constructed from previous studies on the HPA axis. For example, as shown in Fig. A1, the parameters forming the function $c_\infty(o)$ are derived from fitting to data on adrenalectomized male rats. From the fitting, we estimate the baseline level

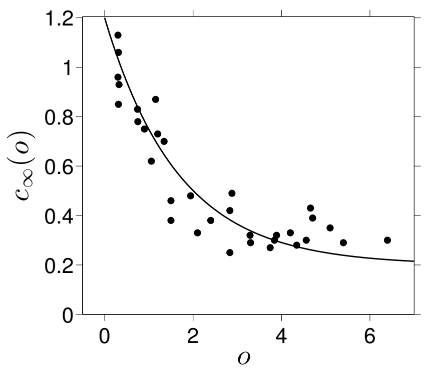

Figure A1: **Fitting $c_\infty(o)$ to rat data.** Nondimensionalized data taken from Watts and fitted using the form for $c_\infty(o)$ given in Eq. 19. Cortisol levels were arbitrarily rescaled according to 125ng/ml $\to$ 3.

$\bar{c}_\infty \simeq 0.2$, and the decay rate $b \simeq 0.56$. Furthermore, the dimensionless parameters

$p_2, \ldots, p_5$ and $t_{\rm d}$ will be fixed to those used in Walker *et al.*: $p_2 = 15$, $p_3 = 7.2$, $p_4 = 0.05$, $p_5 = 0.11$, $p_6 = 2.9$ and $t_{\rm d} = 1.44$ ($T_{\rm d} = 15$ min). Although it is not possible to determine all of the remaining parameters from data, we will use reasonable estimates. The half-life of cortisol was estimated to be about 7.2min while the half-life of CRH has been estimated to be about 4min. Therefore, $q_2 = d_C d_O^{-1} \approx 1.8$. Of the remaining parameters $(n, \mu_{\rm c}, q_0, q_1, k)$, the dependence on n will turn out to be quantitative so we henceforth set $n = 5$. These estimated parameters are listed in Table A1.

Even though one expects the values of these effective parameters to be highly variable, we fix them in order to concretely investigate the mathematical structure and qualitative predictions of our model. The parameters $\mu_{\rm c}, q_0$, q_1, and k remain undetermined; however, it is instructive to treat k as a control parameter and explore the nullcline structure in $\mu_{\rm c}, q_0, q_1$ parameter space.

Parameter space and nullcline structure

To determine how the q-nullcline crosses the c-nullcline, we substitute $c_{\rm s}$ by its equilibrium period-averaged value $\langle c_\infty(c)\rangle$. If we assume a basal input level $I = 1$, the values of k that will position the basal q-nullcline to just pass through the left and right bifurcation points $(q_{\rm L}, c_{\rm L})$ and $(q_{\rm R}, c_{\rm R})$ can be found by solving $q_{\rm L,R} = q_0(1 - e^{-k\langle c_\infty(c_{\rm L,R})\rangle})$:

$$k_{\rm L} = \frac{1}{\langle c_\infty(c_{\rm L})\rangle} \ln\left(\frac{1}{1 - q_{\rm L}/q_0}\right), \quad k_{\rm R} = \frac{1}{\langle c_\infty(c_{\rm R})\rangle} \ln\left(\frac{1}{1 - q_{\rm R}/q_0}\right). \tag{A3}$$

All possible ways in which the nullclines can cross each other as k is varied are illustrated in Fig. A2.

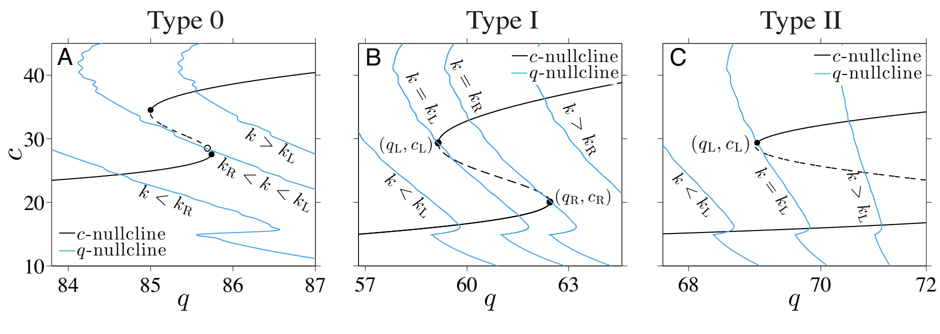

Figure A2: **The possible number of equilibria of the reduced (q, c) system.** (A) A **Type 0** scenario in which $k_{\rm R} < k_{\rm L}$ permits only one nullcline intersection, either one the lower stable branch, the unstable branch, or the upper stable branch. (B) In this **Type I** parameter regime, the c-nullcline is shaped and positioned such that $k_{\rm L} < k_{\rm R}$. Therefore, it is possible for the model to exhibit two oscillating stable states provided $k_{\rm L} < k < k_{\rm R}$. For $k < k_{\rm L}$ ($k > k_{\rm R}$), the q-nullcline shifts to the left(right) and the intersection with the upper(lower) branch of the c-nullcline disappears, leading to only one stable point. (C) A **Type II** c-nullcline. For $k < k_{\rm L}$, there is only one intersection at the lower branch. For all $k > k_{\rm L}$ there are two intersections.

The specific locations of the bifurcation points, as well as k_{L} and k_{R}, are complicated functions of all parameters. However, Eqs. A3 allows us to distinguish three qualitatively different regimes. The first possibility is $k_{\mathrm{L}} > k_{\mathrm{R}}$, where there can be at most only one intersection between the slow and fast nullclines. We denote this as a **Type 0** scenario (Fig. A2A) characterized by having at most a single stable state towards which the system will always return upon cessation of external stress. In Type 0 situations with intermediate values of k, the intersection will arise in the unstable branch of the c-nullcline. In this case, we expect the system to oscillate between the two stable branches of the c-nullcline. Here, the fast variables a, o, and r will cycle periodically between two oscillating levels.

In order for the two nullclines to intersect three times (twice on stable branches of the c-nullcline), the q-nullcline must "fit" within the bistable region of the c-nullcline. As shown in Fig. A2, there are two separate subcases of nullclines that intersect twice. If $k_{\mathrm{L}} < k_{\mathrm{R}}$, a value of $k_{\mathrm{L}} < k < k_{\mathrm{R}}$ would imply that the $q-$nullcline can intersect both stable branches of the c-nullcline, leading to two stable solutions. We refer to this case as **Type I** (Fig. A2B).

Another possibility is that the right bifurcation point is beyond the maximum value $q = q_0$ dictated by the function $h(\langle c_\infty(c_{\mathrm{R}})\rangle)$. As shown in Fig. A2C, the bistable c-nullclines exhibits only one bifurcation point within the domain of q. The lower branch of the c-nullclines in this set extends across the entire range of physiological values of q, ensuring that the q-nullcline will intersect with the lower branch for any value of k. Therefore, to determine if there are two intersections we only need to check that $k_{\mathrm{L}} \leq k$ is satisfied. In this **Type II** case, the system is either perpetually in the diseased low cortisol state, or is bistable between the diseased and normal states; the system will always be at least susceptible to low-cortisol disease. Summarizing,

- **Type 0**: Exactly one solution (one nullcline intersection) exists for the reduced subsystem. Here, $k_{\mathrm{R}} < k_{\mathrm{L}}$ and the intersection may occur on the lower or upper stable branches, or on the unstable branch of the c-nullcline. The system is either permanently diseased, permanently resistant, or oscillates between normal and diseased states.
- **Type I**: At least one solution exists. A stable diseased solution exists if $k < k_{\mathrm{L}}$, two stable solutions (diseased and normal) arise if $k_{\mathrm{L}} \leq k \leq k_{\mathrm{R}}$, and fully resistant state arises if $k > k_{\mathrm{R}}$.
- **Type II**: At least one solution exists. A stable diseased state arises if $k < k_{\mathrm{L}}$ while both diseased and normal solutions arise if $k > k_{\mathrm{L}}$. A fully disease-resistant state cannot arise.

With the parameters fixed according to Table A1, we will treat k as a control parameter and exhaustively sweep the three-dimensional parameter space $(q_0, q_1, \mu_{\mathrm{c}})$ to determine the regions which lead to each of the nullcline structural types. In addition, we restrict the parameter domain to regions which admit oscillating solutions of the full problem. In other words, parts of both stable branches of the c-nullclines must fall within values of c which support oscillations in the PA-subsystem (Fig. 3). The regions in $(\mu_{\mathrm{c}}, q_0, q_1)$ space that satisfy these conditions and that yield each of the types of nullcline crossings are indicated in Fig. A3.

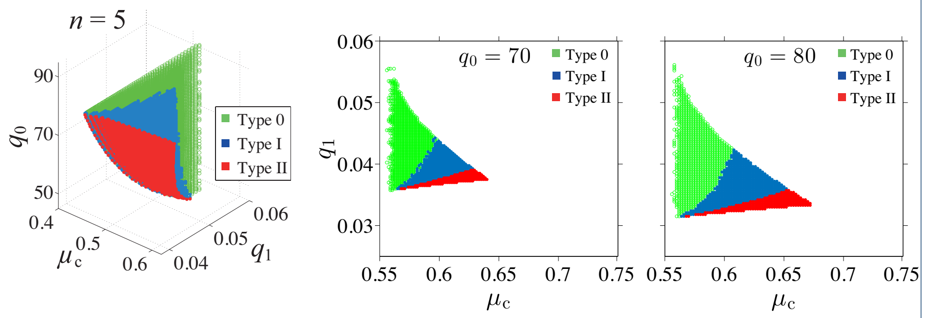

Figure A3: **Phase diagram in** (μ_c, q_0, q_1)**-space.** Regimes for each of the three types of bistable c-nullclines shown in the parameter space (μ_c, q_0, q_1) and (μ_c, q_1) with $n = 5$. The uncolored regions correspond to systems that do not exhibit either bistability or oscillations.

Based on measurements of self-upregulation of CRH secretion during stress, $\mu_c = 0.6$ is chosen to set the baseline level of the Hill function $g_c(c = 0) \approx 0.4$. q_1 is approximated by setting the inflection point of $g_c(c)$ to arise at $c \approx 25$, the average value used by Walker *et al.* Assuming $c \approx 25$ is a fixed point of Eq. 15 when $I = 1$ and $c_s \approx \langle c_\infty(25) \rangle$, q_0 can be estimated as a root of the right-hand-side of Eq. 15. This choice for the remaining parameters puts our nullcline system into the **Type I** category that can exhibit one or two stable states with oscillating (a, o, r) subsystems. We restricted the analysis of our model to **Type I** systems.

Minimum duration and magnitude of stress

We plot the minimum duration required for normal-to-diseased transition against stress magnitude (Fig. A4). Higher magnitude of I_{ext} generally requires a shorter duration of stress, as expected. Note that the minimum duration is also dependent on the phase of intrinsic oscillations of the system at stress onset.

Timing of stress onset and transient response

Here, we show how the dynamics of the system changes after the onset and cessation of stress. In previous studies, changes in corticosterone levels in rats weremeasured in response to stress induced by noise applied at different phases of the animals oscillating cortisol cycle. It was observed that the timing of the stress onset relative to the ultradian phase was crucial in determining the magnitude of corticosterone response. Increases in corticosterone levels were markedly higher when noise was initiated during the rising phase than when initiated during the falling phase.

We can frame these experimental observations mechanistically within our theory. Following the experimental protocol, we simulate the stress response using a brief stressor with a duration of 30min. As shown in Fig. A5A, an external stress that is applied mostly over the falling phase of the cortisol oscillation results in a higher subsequent nadir in $o(t)$ than one that is applied predominantly during a rising phase. However, as shown in Fig. A5B, stress applied mainly during the rising phase leads to a higher subsequent peak level. This observation is consistent with

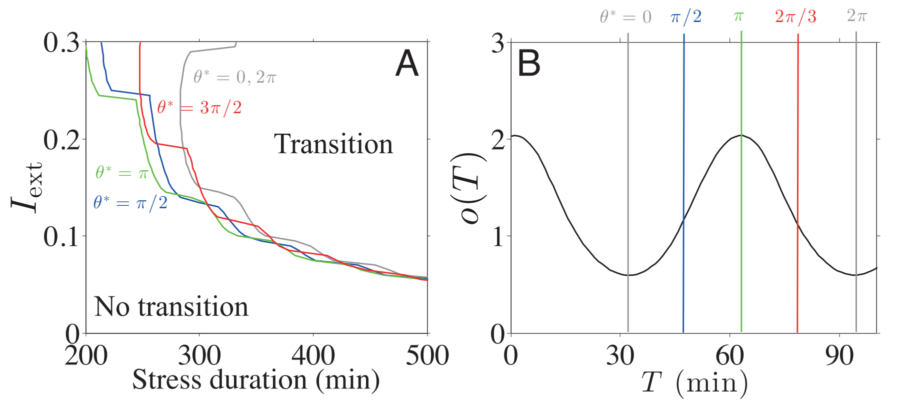

Figure A4: **Phase diagram of stress-induced transitions** (A) Minimum duration of stress required for normal-to-disease transition is plotted against stress magnitude. The phase of intrinsic oscillations at stress onset is denoted as θ^*. (B) Four θ^* values were chosen and marked on the plot of $o(T)$ with different colors assigned. The color of each curve in plot (A) corresponds to the θ^* of stressor onset shown in (B).

the results of the experiment on rats and can be explained by the dynamics inherent in our model.

The immediate increase in $q = q_0 I h(c_{\rm s})$ associated with the increase in I leads to a rapid increase in c, as shown in Fig. 7. This higher level of circulating CRH shifts the stable limit cycle of the PA subsystem to a new one with higher minimum and maximum values of ACTH and cortisol (as shown in Fig. 3). This new limit cycle is shown by the blue curve in Figs. A5C,D. Under external stress, a trajectory of the system quickly deviates and approaches the new limit cycle, but quickly returns to the original limit cycle after cessation of stress. Thus, depending on the position of the trajectory relative to that of the new stressed limit cycle, the initial deviation may try to reach the new limit cycle in the falling or rising cortisol phases as shown in Figs. A5C,D. Moreover, if the duration of the stress is shorter than the period of the inherent oscillation, the trajectory will return to its original limit cycle before completing a full cycle of the new limit cycle. These properties of the limit cycle dynamics explain the difference in the level of subsequent peak following the stress onset depending on the timing of the stress onset.

Cortisol-dependent $I_{\rm ext}$

Since it has been shown that synaptic input into PVN cells is modulated by cortisol for certain stressor types, we briefly discuss how cortisol-dependent $I_{\rm ext}(T, O)$ may affect results from our model. We assume a simple form of $I_{\rm ext}(T, O) = I_{\rm time}(T) + I_{\rm cort}(O)$ where $I_{\rm ext}(T, O)$ assumed to be lower when cortisol levels are higher. A possible form of cortisol-dependent $I_{\rm ext}$ is illustrated in Fig. A6B. Since cortisol does not affect the basal release rate, the cortisol-dependent component of the external input function, $I_{\rm cort}(O)$, should be zero when there is no stress. On the other hand, it was also shown that the inhibition effect cannot decrease the release rate below the basal rate so we can further assume that $I_{\rm ext}(T, O) \geq 0$. When these conditions are met, the modification in $I_{\rm ext}(T, O)$ should not affect the bistability of the system since $I(T) = I_{\rm base} = 1$ is unchanged. However, a cortisol-dependent $I_{\rm ext}(T, O)$

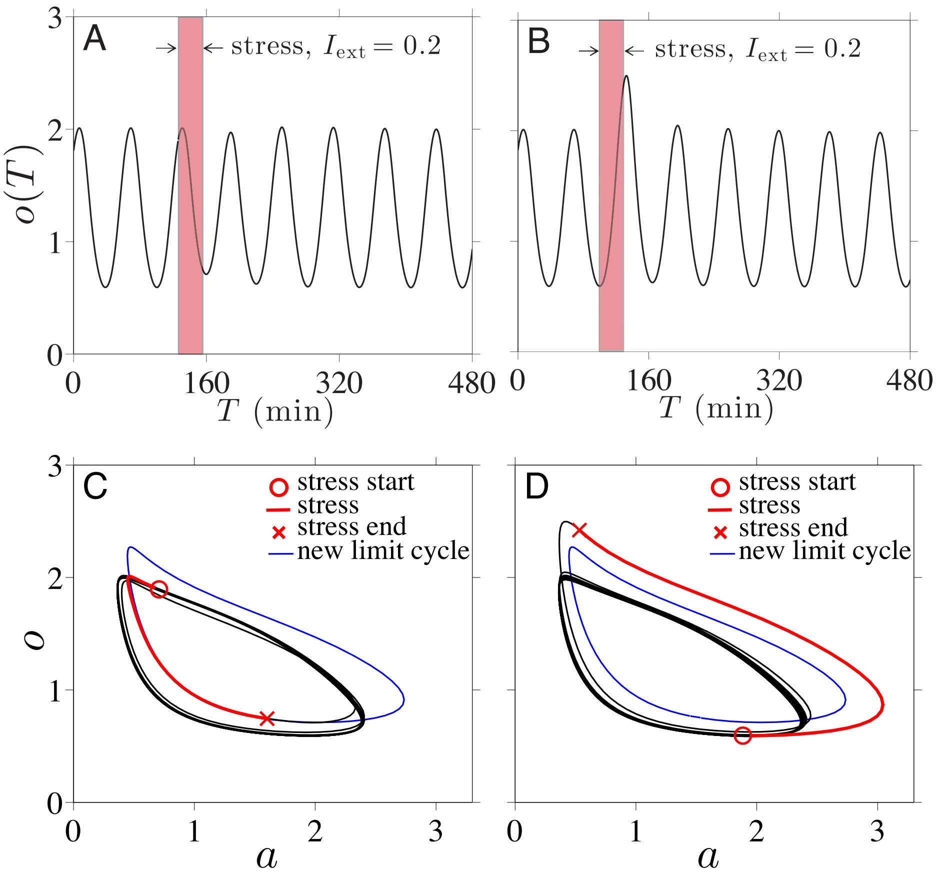

Figure A5: **Stress timing and cortisol response.** (A) A stressor of duration of 30min with magnitude $I_{\text{ext}} = 0.2$ was applied mainly over the falling phase of the underlying cortisol oscillation. The first peak after the stress onset was almost unchanged, but the first nadir was elevated. (B) The same stressor used in (A) applied during the rising phase led to a significantly increased subsequent peak while the first nadir was unaffected. (C) The trajectory of the system (red) is projected onto the cortisol-ACTH plane. The new limit cycle of the PA-subsystem corresponding to fixed $I(t) = 1.2$ is indicated by the blue curve. During stress, the trajectory of the system is attracted towards the new limit cycle. The system recovers after making a smaller cycle within the normal limit cycle, reaching a higher nadir. (D) The trajectory of the system deviates then recovers back through a trajectory above the normal limit cycle, reaching a higher peak.

will make the timing of stress onset become more relevant in predicting whether a stressor can induce transitions between normal and diseased states. Driven by the intrinsic oscillations in $O(T)$, $I_{\text{ext}}(T, O)$ will also oscillate during stress, driving the q-nullcline back and forth in the (q, c)-plane as shown in Fig. A6C. Stress-driven oscillations in the q-nullcline affect the net decrease in q, changing the position of the system on the (q, c)-plane relative to the separatrix between the normal and the diseased basins of attraction upon stress termination. Since transitions are sensitive to the position of q at stress termination, including a cortisol-dependent $I_{\text{ext}}(T, O)$ will make transitions more strongly dependent on the timing of stress onset.

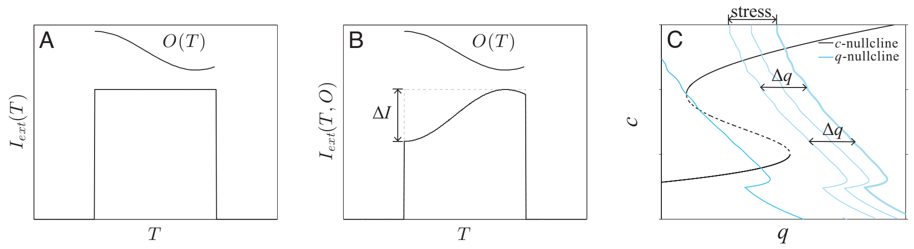

Figure A6: **Cortisol-dependent synaptic input of the PVN and its possible effects** (A) Cortisol independent $I_{\text{ext}}(T)$ used in our current model. (B) An example of cortisol-dependent $I_{\text{ext}}(T, O)$, where we assume the synaptic input of the PVN is attenuated at higher levels of $O(T)$. (C) During stress, the q-nullcline shifts back and forth in the (q, c)-plane due to oscillations in $I_{\text{ext}}(T, O)$ as driven by the intrinsic ultradian oscillations in $O(T)$. In turn, theses shifts in q-nullcline will affect the net decrease in q during stress.
